# Nomen est omen: Investigating the dominance of nouns in word comprehension with eye movement analyses.

**DOI:** 10.2478/v10053-008-0069-1

**Published:** 2009-12-23

**Authors:** Marco R. Furtner, John F. Rauthmann, Pierre Sachse

**Affiliations:** Department of Psychology, University of Innsbruck, Austria

**Keywords:** word class, noun–verb debate, word comprehension, transposed word reading, eye movements

## Abstract

Although nouns are easily learned in early stages of lexical development, their role in adult word and text comprehension remains unexplored thus far. To investigate the role of different word classes (open-class words: nouns, adjectives, verbs; closed-class words: pronouns, prepositions, conjunctions, etc.), 141 participants read a transposed German text while recording eye movements. Subsequently, participants indicated words they found difficult and reproduced the story. Then, participants were presented an untransposed text version while also tracking eye movements. Word difficulty, subjectively assessed by an interview and objectively by eye movement criteria (general fixation rate, number of fixations on specific words), text comprehension scores, and regressive fixations from one word class to another in the transposed text indicated that the noun was the most influential word class in enhancing the comprehension of other words. Developmental, intercultural, and neurophysiological aspects of noun dominance are discussed.

## INTRODUCTION

Language teachers are often confronted with quite a common phenomenon when correcting translation exercises: Nouns seem to be translated adequately; verbs, however, are either omitted or their meaning has been fantasised by the pupils. Pupils “guess” any verb that is most likely to occur with the noun and fit the context. This observation taps the long-lasting debate regarding the universal advantage of either the noun or the verb (e.g., Childers & Tomasello, 2001; Gentner, 2006; Imai et al., 2008; Kersten & Smith, 2002) as the predominant word class, especially in early lexical development. A related question is which word class enhances speech acquisition, language learning, and word comprehension in adulthood. It can be expected that trajectories from early lexical development also affect language, speech, and reading in adulthood. According to Arciuli (2009), the question remains how adults distinguish nouns and verbs in reading. Further, Arciuli concludes that “in particular, there remain a great many gaps in our understanding of lexical representations” (p. 633) which pretty much summarises the current problem in linguistics. 

In this article, we concentrate on adulthood word comprehension and investigate the hypothesis of noun advantage by experimental means. We will show evidence from an eye movement study that the noun can indeed be considered the main semantic element of a phrase. The noun is shown to enhance word comprehension as most information can be drawn from this word class.

## Theoretical background

### Word classes

The classification of different word categories dates back to the ancient times of Aristotle and Dionysius (cf. Baker, 2003; Gentner, 1982). However, to this day there is no universal answer to the question which word class enhances the comprehension of reading and language. Soon, academic psychology – being a relatively young discipline – attended to this topic. Karl Bühler, one of the first modern psycholinguistics, has reported in 1934 about the importance of the verbal world view of Indo-European languages. Yet, down to the present it has not been empirically clarified *which* word class or even *if* a word class enhances the comprehension of words which are difficult to understand. Such a word class would offer an increase of informational content within a phrase. Mostly, nouns and verbs have been analysed with respect to this issue. The findings of previous studies led to intense discussions which word class, nouns or verbs, may have the predominant role in human language (cf. Imai, Haryu, Okada, Li, & Shigematsu, 2006). Dürr and Schlobinski (2006) conclude from their analyses that the most important distinction of word classes concerns nouns versus verbs as this is the only distinction which can be consistently found across different languages. For example, adjectives are in some languages merely attributes to nouns and not counted as a separate, distinct word class.

### Research on different word classes

#### Pro noun

Evidence for the dominance of the noun comes especially from developmental studies concerning the debate on the supposed universal advantage in noun learning. Nouns should be learned more easily in early stages of lexical development “because concepts denoted by nouns are cognitively more coherent and accessible than concepts denoted by verbs” (Imai, Okada, & Haryu, 2005, p. 341; cf. Gentner, 1982, 2006; Gentner & Boroditsky, 2001; Imai et al., 2008). Verbs may not be learned as easily as nouns due to following reasons: First, actions can be more difficult to encode and to remember than objects as “the concepts verbs typically denote (i.e., actions) themselves are more difficult to learn than the concepts nouns typically denote (i.e., concrete physical entities), presumably because actions are less tangible perceptually than concrete physical entities” (Imai et al., 2005, p. 353; cf. Gentner, 1982; Golinkoff, Jacquet, Hirsh-Pasek, & Nandakumar, 1996). Second, the semantic criteria of generalisation vary for different types of verbs (Imai et al., 2005; Maguire et al., 2002). This may also be associated with the fact that verbs require due to their usually enhanced morphology (they make references to implicit subjects, objects, tenses, and modes in their flexion) a greater deal of grammar which is located in the procedural memory, whereas most objects or nouns could be treated as simple facts and would therefore be located in declarative memory. To generalise and use a verb properly (and grasp its semantic meaning in different situations), more experience is required which leads to a third point: Children might not need much experience with objects in order to extend a certain noun to a new instance; the complexity of verbs does not allow such a rather conservative approach as one ought to have accumulated a larger amount of experience in the action which a verb denotes in order to generalise and extend it to other situations (Imai et al., 2005). This is refers to the “physicality” of most nouns: There is a concrete object that can be mentally represented in semantic memory, whereas verbs tend to be abstract and are not accompanied by a certain picture. Object concepts labelled by nouns are related to both visual and action representations; thus, they are easier to learn than action concepts which are labelled by verbs and only related to action representations.

 Gentner (2006) declares nouns as the natural origin of acquiring a new language. She developed the hypotheses of natural partitions and relational relativity: 

(1) There is a universal and early dominance of the noun in speech and language acquisition.

(2) A basis of available nouns helps children understand and learn also less transparent relations among terms (e.g., verbs and prepositions).

(3) By children, new types of nouns are more willingly learned than new types of verbs.

(4) Within the class of nouns, there are concrete objects (e.g., *table*, *grass*, *window*, etc.) that are learned earlier in childhood.

(5) Children need longer to learn the full meaning of a verb.

(6) The processing of verbal meaning influences the way new verbs are learned.

(7) Nouns are also preferred when learning a second language.

 People learning a second language tend to make more mistakes in verbs than nouns (Lennon, 1996). According to Gentner (2006) , the acquisition of verbs in early childhood development lags behind the acquisition of nouns due to following three reasons: (a) the biological process of maturation, (b) difficulties in learning which semantic elements belong to the verb and how these may be combined, (c) the arrangement of information. The sequence “noun before verb” may be seen as a relatively general pattern of speech acquisition. Concrete nouns connect in a more transparent way than verbs. 

 An exceptional study was conducted by Badalamenti (2001) : He evaluated the occurring frequency of nouns, verbs, adjectives, and conjunctions in texts. The first 5,000 words of the works from four popular authors, Chaplin, Shelley, Twain, and Smith, were analysed this way. Results indicate that nouns are the only word class that do not show any significant differences in occurring frequency variation throughout the texts: The frequency distribution of nouns remains relatively constant throughout all the works. Hence, the author presumes a fundamental and exceptional position of the noun for the structure of written English language. Also, the noun contributed to organising and structuring the texts and provided the largest amount of information for each author. The nouns served as a basis for different usage variations of other word classes (verbs, adjectives, etc.). 

#### Pro verb

Intercultural studies concerning speech acquisition and linguistic usage do not always show a predominant position of nouns. Equally frequent usage of nouns and verbs is reported for Korean (Choi, 1998) and for Mandarin even more frequent usage of the verb (Tardif, 1996). According to studies of Camaioni and Longobardi (2001) , Italian mothers produce more verbs than nouns while talking to their children. The authors also point out the semantic and morphologic significance of verbs compared to nouns. On the other hand, an intercultural study with regard to Spanish, Dutch, French, Hebrew, Italian, Korean, and English (Bornstein et al., 2004) shows a higher frequency of nouns considering the available vocabulary of children. Further, every word class was positively correlated with its particular counterpart in the other languages. The level of differentiation between noun and verb also plays a significant role in speech acquisition and production: The difference between a noun and verb can be more easily assessed in German and Dutch than in English. Also, there is a difference in verb position in different languages, depending on the grammatical structures. English is referred to as a verb-second language (SVO-language: subject – verb – object; e.g., *Bill* [S] *didn’t buy* [V] *any fish* [O].) and German as well as Dutch as verb-final languages (SOV-languages: subject – object – verb; e.g., *Bill* [S] *hat keinen Fisch* [O] *gekauft*[V].; De Bleser & Kauschke, 2003). 

The “pro verb” positions stem mostly from the fact that both subjects and objects tend to be dropped in Asian languages (e.g., Chinese, Korean, Japanese) and that verbs are more frequently verbalised by mothers (cf. Choi & Gopnik, 1995; Tardif, 1996). However, it should be noted that with regard to the Asian languages there are also mixed results on the proportion of learned nouns and verbs indicating either a dominance of the noun (Au et al., 1994; Kim et al., 2000) or the verb (Choi, 1998). Also, it has been often found that novel noun learning is easier than novel verb learning (Casasola & Cohen, 2000; Childers & Tomasello, 2001; Imai et al., 2005; Kersten & Smith, 2002; Werker, Cohen, Lloyd, Casasola, & Stager, 1998).

Fillmore’s (1968, 1969) casus grammar, a theoretical model of generative grammar, also emphasises the importance of predicate-attribute-structures. The verb is usually equivalent to the predicate of a proposition. In Fillmore’s theory, the predicate has in very proposition a regulatory function as it determines how many and which arguments are necessary. The arguments themselves relate to the nouns in the generative grammar. Fillmore distinguishes different types of arguments which can be found in every language and could be innate. An experimental study of Hörmann (1976) emphasises the important regulatory and information-bearing role of the predicate or verb: Participants heard phrases which they were to reproduce after presentation, but this was made difficult by white noise. The verb was then least recognised in all clauses. However if the verb was recognised, then it was more likely that subjects and objects would also be correctly heard. Conversely, correctly recognising subjects and objects did not lead to enhanced recognising of predicates. From this the authors conclude that the verb seems to contain most information in a phrase. 

#### Other word classes

Many studies merely concentrated on specific analyses of the word classes of nouns and verbs (Dürr & Schlobinski, 2006), whereas the role and impact of adjectives and especially closed-class words (e.g., pronouns, prepositions, conjunctions, etc.) has often been neglected. Harley (2001) gives a differentiated description of different word classes: (a) nouns: (concretely or abstractly) naming and designating animate and inanimate objects; (b) verbs: indicators of actions, states, processes, or statements; (c) adjectives: descriptors or attributes of animate and inanimate objects; (d) adverbs: qualifiers of verbs. Nouns, adjectives, verbs, and most adverbs are referred to as *content words* as they represent the semantics of a language and convey meaningful content; closed-class words, on the other hand, mainly structure the grammar of a language. As closed-class words are quite seldom newly added or adopted to a language, they are referred to as a *closed class of words*. 

According to Ferrer i Cancho and Solé (2001), closed-class words play only an insignificant role in text comprehension. Multiple studies are concerned with the commonalities and differences between content and closed-class words. Schmauder, Morris, and Poynor (2000) state that closed-class words contain less semantic content. Studies show that closed-class words are faster accessible than content words, indicating that the processing of often used closed-class words takes place in quite a short time. After this first and rapid processing the focus of attention is allocated to the semantically meaningful elements, the content words. In general, an increase in the frequency of using a certain word is accompanied by an increase in cerebral processing mechanisms of that word (cf. Furtner & Sachse, 2007). Yet, there is a difference between equally often used closed-class and content words: Closed-class words are in relation to content words more frequently omitted during reading (Greenberg, Healy, Koriat, & Kreiner, 2004; Roy-Charland & Saint-Aubin, 2006). A further difference between closed-class and content words occurs in higher word frequency and less word length of closed-class words within a phrase (Rayner, 1998). 

Closed-class words are linked to the left anterior region of the human brain; automatised and rapid language processing of frequently used words takes place especially in this area (Segalowitz & Lane, 2004). The lexical access to closed-class words is located in the perisylvian region, whereas content words additionally involve other regions of the brain depending on the specific meaning of a word (Pulvermüller, 1999). The findings of Schmauder and colleagues (2000) could not support the thesis that closed-class and content words are represented in different lexical units.

### The present study

#### Aims and scope

The present study replicates and extends previous findings in following ways: First, it is shown that nouns hold more information and can thus be considered as predominant in word comprehension. Second, the fact that there is an advantage of the noun in early lexical development should entail trajectories into adulthood: Adults should be more noun-fixated and use nouns as “semantic anchors” in understanding texts (even though this is an implicit process). This is demonstrated in the present study. Third, experimental methods (eye-tracking analyses, transposed letters) are used to explore word comprehension. Fourth, analyses do not merely focus on nouns and verbs while neglecting other word classes. For example, the role of adjectives (relative to other word classes) remains poorly understood as of yet. To the best of our knowledge, no studies have so far investigated the topic of word classes and word comprehension with our methodological approaches. The present article thus shows that early lexical development still influences reading processes in adulthood in the sense that one retreats to nouns when aiming to better comprehend a difficult word or text. Moreover, the noun–verb debate is further investigated with a novel methodological approach, and the studies’ findings corroborate the noun’s predominant position.

#### General hypotheses

In mind of the current debate on whether nouns or verbs be considered the predominant and most semantic information-holding word class, following main question was the starting point for the study presented here: Which word class (content words: noun, verb, adjective; function words: pronouns, prepositions, conjunctions, etc.) substantially enhances word comprehension?

Based on the literature, two general hypotheses were generated which were to be tested by an experimental design employing eye movement analyses and jumbled (transposed) versus unjumbled (untransposed) text reading (Grainger & Whitney, 2004) in both studies. First, it was hypothesised that the more often refixated word class is used to improve the understanding of words. Participants should significantly more refixate to semantically important words while reading, especially if the whole sentence is hard to understand (due to transposed words). This, in turn, should enhance word understanding. Keeping in mind the early noun advantage (e.g., Gentner, 2006), the noun, in relation to other word classes, was hypothesised to be significantly more refixated to while reading. Second, it was hypothesised that closed-class words generally play a negligible role in text comprehension (Ferrer i Cancho & Solé, 2001).

#### Investigation methods and specific hypotheses

 The combination of transposed word reading and eye-tracking in studying predominant word classes is a novel approach. The method of transposed or “jumbled” words was introduced by Grainger and Whitney (2004) for the first time. Studies with transposed words and texts have shown that participants can still read and understand the texts quite well (Rayner, White, Johnson, & Liversedge, 2006). Results from early eye-tracking studies in this field show that the difficulty in understanding a word is dependent on (a) how strongly the word is transposed, and (b) how familiar the word is (e.g., Perea & Lupker, 2004). Additionally, Rayner and colleagues (2006) confirmed that readers especially show more and longer gaze fixations when being confronted with difficult (and unfamiliar) words. The method of transposing words served to partially control contextual effects, provide a difficulty in reading that is not attributable to unknown and/or bizarre words, and account for individual differences in linguistic abilities and adeptness that would both occur too strongly in a “normal” text. Two hypotheses were generated for this study. 

Hypothesis 1: There is a significant difference between the transposed and untransposed text concerning mean fixation durations. That is, the transposed text should need longer to read because the automatic process of reading, which entails skipping of many words (Rayner, 1998), cannot be applied and words need to be mentally untransposed while reading in order to understand them. There should be more difficulty in reading which is reflected in longer gaze times.

Hypothesis 2: The noun is the predominant word class in enhancing word comprehension. This rather global hypothesis is tested by showing significant differences between nouns and other word classes concerning regressions: Confronted with a word difficult to understand, people should regress more often to nouns in order to make sense of the phrase. People would usually not regressively fixate already read words (e.g., nouns) if there was not the need to cognitively infer (more) meaning from the regressed words because there are difficulties in understanding other words (e.g., verbs).

## Methods

### Participants

In our study 141 students participated, out of which 91 were women (64.5%) and 50 men (35.5%). Mean age was 24.6 years (*SD* = 5.00, range: 13-49 years). All participants were capable of reading the presented stimuli either by normal eye sight or by corrected-to-normal vision. Most students were studying psychology at the Leopold-Franzens University of Innsbruck (Austria) at the time of the experiment. The native language of all participants was German. The participants had neither any prior knowledge to the purpose of the experiment nor have they been in previous contact with the stimulus material (which was asked after the experiment).

### Stimulus material

**Figure 1.**
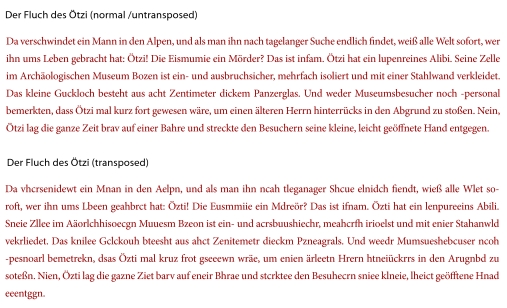
Sequence German text “Der Fluch des Ötzi” in two versions (normal /untransposed, transposed).

The first paragraph of a German text, “Der Fluch des Ötzi” (English: “Ötzi’s Curse”) with 103 words was presented in a transposed and untransposed version (Michel, 2004; see Figure 1). Both text versions were presented left-aligned with the TrueType-font Times New Roman in font size 34 with a line spacing of 1.5.

The text was diligently chosen by following criteria: (a) distribution of word classes common for normal German texts, (b) not difficult in words and context, (c) very likely to be unknown to prospective participants, (d) rather short.

Context effects are a very strong source of variance in word comprehension tasks: Initial, rather unknown, and/or bizarre words may be more of an “eye-catcher” and thus might be fixated more. To control these effects, all words were transposed with a special algorithm by the software programme “Der Wortverdreher” by M. Hahn1 (English: “The Word Jumbler”), which uses two specific rules of randomly jumbling letters (Rule 1: The first and last letter of a word remain untransposed; they stay on their initial and final positions. Rule 2: If a word contains only two or three letters, that is, it is monosyllabic, then it remains untransposed as it cannot be jumbled without breaking Rule 1.) Therefore, only the middle letters of polysyllabic words are transposed. The transposed letter design was also to ensure that the comprehension of nearly all words is not immediately given as they need to be cognitively unjumbled (which is, however, a rather automatic process). This should also control individual differences in word fluency, word knowledge, and general linguistic abilities (even though linguistically more adept people might still be able to read jumbled texts faster).

### Apparatus

**Figure 2.**
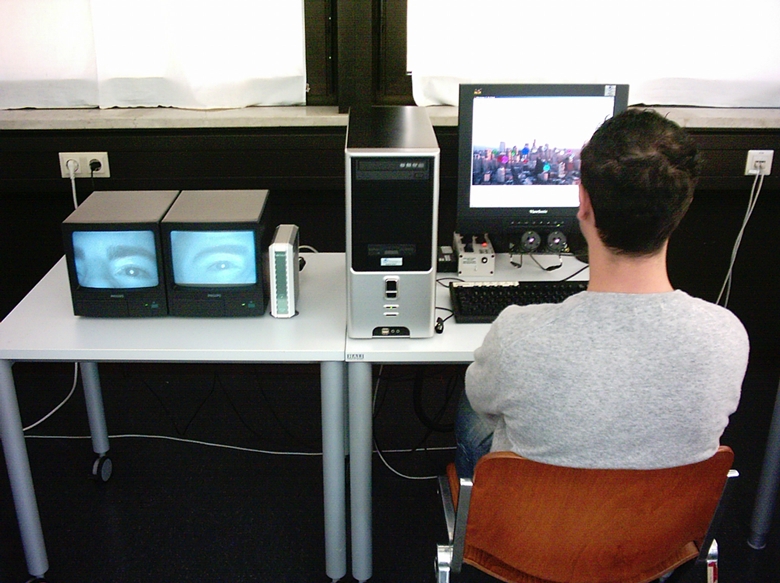
Participant during eye-tracking experiment.

A Pentium IV computer with a graphics card NVIDIA GeForce 4 MX 4000 was used. The German text was displayed on a 17-inch computer monitor (View Sonic VG700b) with a display refresh rate of 75 Hz. Eye movements were recorded with a frequency of 2 x 60 Hz with two binocular cameras which were positioned beneath the computer display. The software of the Eyegaze Analysis System from LC Technologies Inc. was NYAN which allowed registering, recording, and analysing fixations (point between two saccades in which eyes are relatively stationary and information input occurs; range from 100 to 1,000 ms) and saccades of participants (see Figure 2). Two observation monitors allowed watching the right and left eye (through input from the left and right binocular camera beneath the computer display) while in the process of eye-tracking in order to correct the sitting posture of participants if necessary.

### Experimental setting and procedure

#### Step 1: Eye-tracking with transposed text

First, the eye-tracking device was calibrated to fit the individual eye movement patterns of the participants which took on average about 3 min. After successfully calibrating, the actual presentation of the transposed German text (see Figure 1) began and the participants were instructed to specifically concentrate on understanding the text. The text was displayed as long as participants needed to read the whole text which took on average about 3 to 4 min. All participants were neither familiar with the stimulus material nor did they know about the four steps of the experiment and what they had to do in each of them.

#### Step 2: Indication of difficult and incomprehensible words

Subsequently to the stimulus presentation, the participants were asked to name the most difficult and incomprehensible words. The transposed text was again displayed via MS PowerPoint, and participants could indicate the words that they found difficult. The experimenter simultaneously marked for each participant the most difficult and incomprehensible words on the monitor.

#### Step 3: Reproduction of transposed text content (comprehension)

Then, the text was removed and participants were questioned about their content-related text comprehension (i.e., what they actually understood from the text). The participants were to orally reproduce the story from their memory. The experimenter had a checklist of potential content blocks (see Table 1) that could be reproduced. For each block named by a participant (i.e., if a participant made somehow reference to it while reproducing the story) he or she obtained one score point. Then, sum scores of comprehension can be computed for each participant. This step was very important as the instruction in Step 1 to concentrate on word comprehension ensured that participants would actually read the text and not just look at the words without processing any information. Note that eye-tracking does not allow us to directly draw conclusions about underlying information processing processes. That something is focused (i.e., fixated) is not necessarily indicative of attention or occurring information processing (cf. Mack & Rock, 2000).

**Table 1.** Content Block Checklist for the Reproduction

Content blocks	Item 1	Item 2	Item 3	Item 4
Content block 1	*man disappeared*	*found dead*	*Ötzi the murderer?*	–
Content block 2	*museum Bozen*	*vbooth steel wall*	*spy hole armoured glass*	–
Content block 3	*Was Ötzi gone?*	*elderly man down the precipice*	*Ötzi in his bier*	*Ötzi’s hands reaching out*

*Note*. Participants were to orally reproduce the transposed word text and their answers were marked on a checklist consisting of ten minor content blocks (in three major content blocks) derived from mean-ingful units of the text.

#### Step 4: Eye-Tracking with normal text

Then, the eye-tracking device was again calibrated to ensure accurate recording of eye movement parameters for a second eye-tracking procedure. The text was then presented in its “normal” and untransposed version to the participants. The text was displayed as long as participants needed to read the whole text which took on average about 1 to 2 min. The whole experiment took about 12-15 min.

### Data preparation and statistical analyses

#### Preliminary analyses of stimulus material

First, the content of the German “Ötzi” text was analysed for content words (nouns, verbs, adjectives) and closed-class words (pronouns, prepositions, conjunctions, etc.). An analysis of word class frequency shows following distribution: The text contains 26% (*n* = 27) of nouns, 17% (*n* = 18) of adjectives, and 15% (*n* = 15) of verbs, that is 58% of content words, and 42% (*n* = 43) of closed-class words, with 103 words in total. Although there is a mismatch between the different content words in their absolute numbers, this distribution resembles quite a common distribution in German texts (nouns: 46%; verbs: 19%; adjectives: 23%; adverbs: 6.7%; prepositions: 1.2%; conjunctions: 1.3%; pronouns: 0.8%; see Drosdowski, 1995). It would not have been ecologically valid to present a text that contains, for example, more verbs than nouns because this simply is seldom the case.

#### Analyses of Steps 1 and 4 (eye-tracking data)

**Figure 3.**
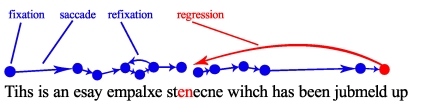
Fictitious example of the specific regressions of a word difficult to understand.

Both texts, untransposed and transposed, were analysed with respect to the mean fixation duration (in milliseconds) by a *t*-test for dependent means as the experiment uses a within-subject design. Concerning the main question of this study to assess which word class substantially enhances word comprehension, specific regressive fixations of participants were analysed (see Figure 3). For instance, to help the comprehension of the difficult transposed word *jubmeld*, it is first compared with the preceding transposed noun stenecne (regression) and then eye fixations recur from this comprehension helping noun to the difficult word again. Sixteen different types of regressive fixations were possible considering the combination possibilities of content words (nouns, verbs, adjectives) and closed-class words (see Table 2).

**Table 2.** Possible Combinations of Regressions

		Regression to...			
		Noun	Verb	Adjective	Closed-class word
Word classes difficult to understand	Noun	x	x	x	x
	Verb	x	x	x	x
	Adjective	x	x	x	x
	Closed-class word	x	x	x	x

#### Analyses of Step 2 (word difficulty)

The difficulty of word comprehension was assessed by three criteria to account for subjective word difficulty by a qualitative method and objective word difficulty by two objective methods: (a) interview of participants, (b) general frequency of word fixation in the transposed text, (c) number of fixations by each word. An increase in word fixation is accompanied by an increase in the individually determined difficulty level of a word (Rayner et al., 2006). During the interview (Criterion 1) which aimed at assessing difficult transposed words for participants and thus tapped subjective word difficulty, the transposed text was displayed again so that the participants could identify the critical words. Frequency distributions were computed. To more objectively assess the difficulty of transposed words and not just rely on participant’s statements, also eye-tracking data from the transposed text version was used (Criteria 2 and 3). Relevant eye-tracking literature provides evidence that general fixation frequency and number of fixations can be considered as indicators of word difficulty (see Goldberg & Wichansky, 2003). The more often a word is fixated (and the higher the amount of fixations per word is), the more difficult it is to understand. The fixation frequency was analysed in two ways. First, words were analysed according to their general fixation (not less than three fixations were counted; cf. Rayner, 1998) and then ranked. The question for each word analysed was: How many participants fixated the word more than three times? As a result, the absolute number of participants with more than three fixations per word is obtained. Second, words were analysed according to the number of fixations and then ranked. The question for each word was: How often was it fixated by all participants? As a result, the mean number of fixations is computed.

#### Analyses of Step 3 (text comprehension)

As the participants should not just read but also understand the transposed text, the memory performance of the participants was assessed at Step 3. Scores of the oral reproduction indicating text comprehension were then separated into ten different content-related subcategories of the text (see Table 1). Memory performance according to sex was analysed by a *t*-test for independent means.

## Results

### Analyses of eye-tracking data

#### Hypothesis 1

Mean fixation durations of the transposed text (127 ms) were compared to the untransposed text (114 ms) significantly higher, *t*(125) = 9.67, *p* < .001; *r* = .58; *d_z_* = 0.83, power = 1.00. Thus, the hypothesis that people need longer for reading the transposed text (i.e., they show longer gazing times) is supported.

#### Hypothesis 2

With respect to our question of which word class enhances word comprehension, the regressions in the transposed text were analysed (see Tables 3 and 4): For example, if a transposed noun was difficult to understand, then 48% of regressions were made to another noun to enhance word comprehension, 23% to adjectives, 20% to closed-class words, and only 10% to verbs. The percentage of regression supports the hypothesis that people resort more to nouns than to other word classes when trying to make sense of a given phrase.

**Table 3.** Descriptive Statistics

Difficult word class	Refixated word class	*N*	%	*M*	*SD*
Noun	Noun	75	48	1,40	0,79
	Verb	15	10	1,00	0,00
	Adjective	36	23	1,06	0,23
	Closed-class word	31	20	1,19	0,48
	Total	157	100	1,24	0,61
Verb	Noun	26	49	1,04	0,20
	Verb	6	11	1,00	0,00
	Adjective	6	11	1,00	0,00
	Closed-class word	15	28	1,07	0,26
	Total	53	100	1,04	0,19
Adjective	Noun	89	52	1,40	0,62
	Verb	9	5	1,11	0,33
	Adjective	59	35	1,24	0,43
	Closed-class word	13	8	1,00	0,00
	Total	170	100	1,30	0,53
Closed-class word	Noun	22	42	1,23	0,53
	Verb	13	25	1,00	0,00
	Adjective	9	17	1,11	0,33
	Closed-class	9	17	1,00	0,00
	Total	53	100	1,11	0,38

*Note*. The table presents descriptive statistics for the 16 combinations of "difficult word class – refixated word class" (from Table 2). Percentages of refixated word classes (nouns, verbs, adjectives, closed-class words) that were used for enhancing word comprehension (of either nouns, verbs, adjectives, or closed-class words) are indicated in bold.

**Table 4.** Overall Analysis of Word Classes in the "Ötzi"-Text With Descriptive and *F*-Statistics

Word class	*N*	%	*M*	*SD*	*F*	*p* (two-tailed)
Noun	212	49	1,34	0,65		
Verb	43	10	1,02	0,15		
Adjective	110	25	1,15	0,36		
Closed-class words	68	16	1,10	0,35		
Overall	433	100	1,22	0,53	7,700	0,000

Additionally, a global analysis for the transposed text was employed (Table 4). In about 50% of all cases a noun was used to enhance word and text comprehension if there was a word that was difficult to understand. In about 25% of all cases the adjective (*n* = 110) was used for word comprehension enhancement, followed by closed-class

words with 16% (*n* = 68) and verbs with 10% (*n* = 43). There was a significant difference, F(3, 429) = 7.7, *p* < .001, between word classes which was further investigated by the Games-Howell multiple comparisons post-hoc test (see Table 5): There are significant differences between the noun and all other word classes (noun–verb: *p* < .001; noun–adjective: *p* = .006; noun–closed-class words: *p* = .001).

**Table 5.** Results From Games-Howell Multiple Comparisons (post-hoc-test) From the Overall-Analysis in Table 4

Group I	Group J	Mean difference (I – J)	*SD*	*p* (two-tailed)
Noun	Verb	0,32	0,05	,000	
	Adjective	0,19	0,06	,006
	Closed-class words	0,24	0,06	,001	
Verb	Adjective	–0,13	0,04	,011
	Closed-class words	–0,08	0,05	,361
Adjective	Closed-class words	0,05	0,06	,784

Verbs show a significant difference to adjectives (*p* = .011), whereas none to closed-class words (*p* = .361). Also, adjectives and closed-class words show no significant difference (*p* = .784). These results also support the hypothesis that the noun is predominant in granting access to semantic information which likely increases text understanding.

**Figure 4.**
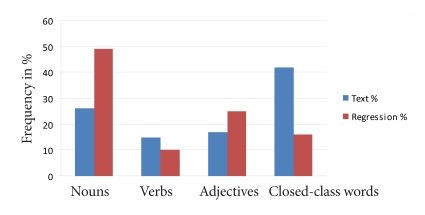
Frequencies of words versus frequencies of regressive fixations for word comprehension enhancement (in percent).

Given the different distribution of word classes within the 103-word text, we employed analyses that take into account the relative percentage of words in the text (58% content words: 25% nouns, 17% adjectives, 15% verbs, 42% closed class) and regressions. The comparison between the occurring frequency of the word classes and their frequency of usage for word comprehension enhancement shows following result: Content words are more frequently used for enhancing word and text comprehension. In total, 84% of all efforts to enhance word and text comprehension were done with content words. Only 16% of regressions were closed-class words. Considering the frequency of word classes and usage of word classes to enhance word comprehension, we obtained the following results (see Figure 4):

(1) There are 26% of nouns in the text, whereas in 49% of all cases nouns are used for word comprehension enhancement (increase: 23%).

(2) There are 15% of verbs in the text, whereas in 10% of all cases verbs are used (decrease: 5%).

(3) There are 17% of adjectives in the text, whereas in 25% of all cases adjectives are used (increase: 8%).

(4) There are 42% closed-class words in the text, whereas in 16% of all cases closed-class words are used (decrease: 26%).

This pattern of findings shows that the noun is the most regressive point in phrases notwithstanding the relative distribution of nouns in the text and is thus convincing evidence for the support of the hypothesis that nouns are predominant in word comprehension enhancement.

Besides the previously mentioned criterion of subjectively perceived difficulty via interview, two more steps via eye movement analyses were conducted to obtain more objective difficulty indices. Specific words were analysed according to their general fixation (not less than three fixations were counted) and then ranked. For example, 99 participants (out of 141) had problems with “Gclckouh” (untransposed: *Guckloch*; English: spy hole) which equalled Rank 4 (see Table 6); 99 participants fixated the word three or more times. Furthermore, specific words were also analysed according to the number of fixations (the mean absolute number of fixations on a specific word across all participants) and then ranked: For example, high number of fixations on an average could be found for “Gclckouh” (untransposed: *Guckloch*), which is on Rank 5 in this category (see Table 6).

**Table 6.** Difficulty Criteria of Words With Descriptive Statistics

Difficult jumbled words	1. Interview		2. General fixation		3. Number of fixations		
	*N*	Rank	*N*	Rank	*M*	*SD*	Rank
*Gclckouh*	105	01	99	04	15,4	11,8	05
*vhcrsenidewt*	88	02	97	05	15,9	06,3	04
*Eusmmiie*	87	03	117	01	17,0	09,7	02
*acrsbuushiechr*	83	04	107	03	17,7	11,5	01
*lenpureeins*	65	05	115	02	16,8	10,0	03
*irioelst*	65	05	65	11	10,0	04,8	14
*meahcrfh*	62	07	78	09	11,2	06,5	09.
*ifnam*	58	08	86	07	15,1	08,0	06
*knilee*	35	09	74	10	09,8	04,6	15
*htneiückrrs*	30	10	83	08	11,7	05,9	07
*Arugnbd*	26	11	62	13	10,7	05,6	12
*Stahanwld*	25	12	48	14	11,6	06,2	08
*tleganager*	23	13	63	12	11,1	07,1	10
*Bhrae*	20	14	38	16	09,1	04,1	16
*Pzneagrals*	14	15	89	06	10,8	06,4	11
*pesnoarl*	13	16	43	15	10,1	03,9	13
*lheict*	13	16	33	17	07,2	05,6	17

*Note*. Difficulty criteria of words: (1) Interview of participants (subjectively perceived difficulty); (2) Fre-quency of general word fixation (question: "Was the respective word fixated three times or more?" Yes = 1, No = 0; N is the index for the absolute number of participants that fixated the respective word three times or more, i.e. obtained in the eye movement analyses from the jumbled text a "Yes"); (3) Number of fixations (obtained from the eye-tracking data from the jumbled text in Step 1).

Subsequent to these analyses, the ranks of the three different criteria were correlated with each other with Spearman’s ρ in order to see how the different criteria related to each other. The highest correlation (ρ = .88) was found among the word ranks of Criterion 2 (general fixation) and 3 (number of fixations) which were both obtained from eye-tracking data. Then follows the correlation (ρ = .81) among the word ranks of Criterion 1 (interview) and 3 (number of fixations); finally, there is the correlation (ρ = .77) among word ranks of Criterion 1 (interview) and 2 (general fixation). All correlations were significant at *p* < .001. Although all correlations were rather high and covered a lot of variance (range from 59 to 77%), the two objective Criteria 2 and 3 related to each other more strongly than the subjective Criterion 1 with either one of the two objective criteria. Given these admittedly rather small but notwithstanding existing differences, it is advisable to not just collect data from either a qualitative or quantitative criterion but rather from both in order to complement each other and form a broader picture in a method-mix analyses.

### Analyses of text comprehension

Participants were able to reproduce 52% of all information of the transposed text on average. No significant difference, *t*(121.429) = 0.49,

*p* = .624, was detected when assessing memory performance in relation to sex. At an average, two items (see content blocks in Table 1) were memorised, whereas the subject areas contained each three or four items.

## Discussion

To answer the question which word class substantially enhances word comprehension, a paragraph out of a German article was presented in an experimental setting in which a unique combination of two methods from cognitive psycholinguistics was used: Transposed word reading and eye-tracking. The eye movement analyses showed that participants recurred with their fixations to certain words when confronted with difficult transposed words. These words were a priori separated into content words (nouns, verbs, adjectives) and closed-class words (pronouns, prepositions, conjunctions, etc.) to evaluate which word class was refixated more often to improve word comprehension.

Results of the study indicate that in 49% of all cases the noun is used for enhancing the comprehension of difficult transposed words (i.e., participants recur with their fixations to nouns). This is a redoubling of the likelihood to refixate to a noun as there are only 26% of nouns in the text. Further, the noun differs significantly from all other word classes as far as frequency of usage is concerned. Compared to the relative frequency of adjectives in the text, there is also an increase in their usage to help comprehend the text better (increase: 8%). Verbs take in a relatively insignificant role if they are compared to the comprehension enhancement by other word classes. This should be seen in comparison to the closed-class words which are used in 16% of all cases for word comprehension enhancement and with 42% of relative frequency within the whole text.

Hypothesis 1 that people need longer for reading the transposed text was supported. Participants showed significantly higher mean durations of fixations while reading the transposed text. People may need longer because the transposed text cannot be read that easily; words have to be cognitively untransposed in order to read the text sensibly. This finding is consistent with Rayner and colleagues (2006) who found that readers show more and longer gaze fixations when being confronted with difficult words. This also elicits an advantage of the transposed-letters paradigm we employed: Research suggests that many words are not fixated in normal reading which, in turn, makes it difficult to focus on regressive fixations as indicators of comprehension enhancement. Transposed letters, however, achieve that people show more fixations as they have to read the text in an unusual way and thus very carefully. Additionally, the design of the experiment may have increased the difference between the untransposed and transposed text version: As the former was presented first, it is likely that people would be faster when reading the untransposed version which did not differ in content.

Hypothesis 2 was also supported: Despite more nouns being in the text, people regressed more often to nouns to increase their understanding when having difficulties in reading. This finding implies that nouns seem to have, in relation to other word classes, more semantic information which helps better word comprehension. As also other word classes were examined, it was found that closed-class words even exceeded verbs at times which is a remarkable finding given that closed-classed words are believed to only play an insignificant role in word comprehension (Ferrer i Cancho & Solé, 2001). However, this finding should not so much be interpreted as the significance of closed-classed words but rather as the insignificance on verbs: In general, verbs seemed not to provide any information that could be used to enhance word comprehension. An explanation for this finding could be trajectories from early lexical development: Verbs are, at least in Western languages, not that easily learned and generalised to other instances, more abstract and perceptionally not concrete (i.e., there is no visual representation of a verb), and require more grammar (e.g., Gentner, 1982; Gentner, 2006; Gentner & Boroditsky, 2001; Golinkoff et al., 1996; Imai et al., 2005; Imai et al., 2008; Maguire et al., 2002). People should therefore be more efficient in understanding and using nouns which is also reflected in eye movement behaviour.

Black and Chiat (2003) show evidence in their multi-facetted psycholinguistic model of single word reading that the noun–verb distinction is not just syntactically relevant but also in other domains of representation, such as semantics, phonology, and orthography (see Arciuli & Cupples, 2006). Generally, the noun’s advantages are emphasised in their model as “phonologically, verbs in English tend to have less typical stress patterns than nouns; to be of shorter duration in sentences; and to have fewer syllables“ (Black & Chiat, 2003, p. 231). Our results indicate that the noun has an advantage in enhancing the comprehension of other words and also support the distinction of the noun from other word classes which is line with Arciuli and Cupples (2006) . However, our results are contrary to Fillmore’s casus grammar which posits that the verb has a central role. Also, our results did not support the findings of Hörmann (1976) that the verb holds most information within a phrase. In their study, the verb could enhance the comprehension of arguments (nouns) but nouns, on the other hand, failed to enhance the comprehension of verbs. Our findings indicate an adverse effect: The noun enhances word comprehension but the verb does not. The noun can thus be seen as the word class with most information within a phrase. This interpretation is supported by Bird and colleagues (Bird, Howard, & Franklin, 2000) who posit that the object (noun) is semantically richer than the action (verb). This can be explained by conceptualising the noun as a thing representing an individual physical entity, whereas the verb represents actions and events (which refer to the physical object). Nouns are also better guessed than verbs (Gleitman & Gillette, 1995): Participants should guess in a silent video (a mother was talking to her child) words that have been beeped out. Verbs were identified only 15% of the time, nouns about 50%. Kemp and colleagues (Kemp, Nilsson, & Arciuli, 2009) showed that adult readers were more sensitive to nouns, which is also in line with previous research. Literature and our findings suggest that the verb seems to be more difficult than the noun. Verbs are additionally learned later than nouns which might be due to the fact that nouns refer to physically concrete things whereas verbs are more difficult to grasp in their relational and contextual usage. Not just for children and for adults but also for aphasic people nouns are the “easier” words (Kemp et al., 2009). Interestingly, the verb is the crucial element in the verbocentric valency grammar as it determines a phrase’s structure upon which all other elements are directly or indirectly dependent (cf. Järventausta, 2003). 

Although we used the German language for our research, the results may not be limited to the German language; it is quite possible that languages with similar qualities to German also show noun preference in word comprehension. However, further research on this field, especially intercultural studies, will be needed to draw a bigger picture on this issue. Intercultural studies provide evidence that the acquisition of nouns, particularly in early speech development, plays a significant role compared to the verb and the other word classes (Werning, 2008); the word class of nouns is predominant in speech production (e.g., Salerni, Assanelli, D’Odorico, & Rossi, 2007) and speech comprehension. In another intercultural study over seven different oral cultures, findings showed that 20-months old children have more nouns at their command than any other word class in their vocabulary (Bornstein et al., 2004). According to Goldfield (2000) , nouns are preferred to verbs in speech production of children. This is quite intelligible as a small child will not say *drive* or *driving* upon viewing a vehicle but rather car or *broom-broom* (which is an onomatopoetic appellation for a driving car). Mothers elicit more nouns from their children and also encourage them rarely to produce verbs. Moreover, mothers would rather animate their children to a concrete action than talking about the action. Further results of this study indicate, however, that children understand verbs better than producing them actively in speech. Nouns seem to be preferred to verbs in speech production (Goldfield, 2000), their acquisition seems easier (Imai et al., 2005; Kauschke, Lee, & Pae, 2007), and moreover they are remembered more easily (Mohr, 1992). 

Further, studies in developmental psychology concerned with speech and language acquisition have already been emphasising the importance of the noun when learning a language in early stages of development (e.g., Gentner, 2006). There is a multitude of studies concerning the acquisition, learning, and development of word classes in children by cognitive developmental psychology. De Bleser and Kauschke (2003) point out the parallels of speech acquisition and language progression patterns between children and adults; the results show a clear preference of the noun in both groups. Hence, one could infer that the best way to learn a new language – even in adulthood – could be to take an approach to the nouns. Indeed, many work books for learning foreign languages start off with more nouns than verbs. This might be a very effective way to learn a new language and develop semantic concept nets as early speech and language acquisition is stimulated in a similar way. 

There is also neurophysiological evidence for the distinction between nouns and verbs in information processing. With respect to psycholinguistics and its neighbouring disciplines, there are various recent studies concerning word classes especially in neurosciences and developmental psychology. Specific studies from a cognitive and experimental psychological view are up to now quite scarce, though. Research with functional magnetic resonance imaging (fMRI) indicates different cortical processing centres of nouns and verbs (content words). Nouns that relate to visually perceptible stimuli are represented in the visual cortex areas (mostly occipital lobe) by neuronal activation. Cell conglomerates specifically responsible for action verbs display additional neuronal connections to motoric, premotoric, and prefrontal areas (Cangelosi & Praisi, 2003; Pulvermüller, Lutzenberger, & Preissl, 1999). Generally, nouns are processed and stored in the temporal lobe and verbs in the frontal lobe. Also, neologisms from nouns and verbs occur in these brain areas. These results hold evidence for a separate status of mental processing (Berlingeri et al., 2008; Goldberg & Goldfarb, 2005; Tyler, Russell, Fadili, & Moss, 2001). Neurobiological studies analysing the word classes show that by tendency separate cortical areas are responsible for processing specific word classes (e.g., Koenig & Lehmann, 1996). Evidence for separate information processing of different word classes also comes from aphasia research: For example, Luzzatti, Aggujaro, and Crepaldi (2006) conducted a study with aphasic patients that were impaired in brain regions responsible for either nouns (lesions in middle and left inferior temporal areas) or verbs (lesions in left posterior temporo-parietal and left fronto-temporal perisylvian areas, insula, and basal ganglia). Application of our findings may also be used for knowledge representation and semantic concept nets or whole networks. According to Werning (2008) , nouns fundamentally differ from other word classes since they are more complex and neural networks distribute more over the entire cortex. 

First, we concentrated only on German with transposed words and thus our results are thus far restricted to the German language (and maybe languages near to German such as Dutch). Second, the stimulus material (or rather its physicality) could have evoked noun-centering effects as nouns in the transposed text (see Figure 1) were still capitalised. Also, one needs to take into account that due to the special syntactic relations in German there might be certain spatial distances that can also account for regression effects (and influence fixation likelihoods). On a more theoretical level, however, it becomes debatable whether the automatism of looking at capitalised words is not a function of semantically and/or syntactically meaningful purposes of the noun. Even if the capitalisation might be a crucial factor for regressive fixations, most information can still be retrieved from nouns nonetheless. Germans call a noun also *Hauptwort* which translates literally as *main word* into English, and in English it may also be called a *substantive* referring to its substantive contribution to a sentence. Future research should thus concentrate on (a) other languages than German, (b) on variation in stimulus material (e.g., capitalisation), and (c) on syntactical and contextual effects, in the hope to replicate and extend our findings presented in this article.

With a combination of eye-tracking and transposed word reading we were able to experimentally demonstrate that the noun can indeed be seen as the predominant word class in word comprehension. The noun enhances word and text comprehension and is preferably used to refixate to when confronted with difficult words. Our findings are in accordance with previous literature on language acquisition (e.g., Gentner, 2006) which also presumes an early dominance of the noun, which is probably still extant in adulthood reading and semantic processes. Various sources (e.g., Black & Chiat, 2003) report a dominance of the noun which our study could corroborate and extend to transposed word reading and word comprehension enhancement. For language acquisition and comprehension our data would suggest that one should start with learning nouns (as opposed to verbs or other word classes) in a foreign language to-be-learned as these can be used to infer (more) meaning from phrases. Nouns can be seen as a semantic central point from which other words can be easier understood. Moreover, our study may also further insights on language comprehension as the noun occupies an important role in most Indo-European languages today. Thus, exploring the processes that underlie word/text comprehension via eye-tracking, also in other languages than in German, can prove fruitful for further research that will hopefully even further advance our knowledge on language comprehension in general.
